# Optimization of Task Allocation for Collaborative Brain–Computer Interface Based on Motor Imagery

**DOI:** 10.3389/fnins.2021.683784

**Published:** 2021-07-02

**Authors:** Bin Gu, Minpeng Xu, Lichao Xu, Long Chen, Yufeng Ke, Kun Wang, Jiabei Tang, Dong Ming

**Affiliations:** ^1^Neural Engineering & Rehabilitation Laboratory, Department of Biomedical Engineering, College of Precision Instruments and Optoelectronics Engineering, Tianjin University, Tianjin, China; ^2^Academy of Medical Engineering and Translational Medicine, Tianjin University, Tianjin, China

**Keywords:** collaborative brain-computer interfaces, task allocation, division-of-work, common-work, motor imagery

## Abstract

**Objective:**

Collaborative brain–computer interfaces (cBCIs) can make the BCI output more credible by jointly decoding concurrent brain signals from multiple collaborators. Current cBCI systems usually require all collaborators to execute the same mental tasks (common-work strategy). However, it is still unclear whether the system performance will be improved by assigning different tasks to collaborators (division-of-work strategy) while keeping the total tasks unchanged. Therefore, we studied a task allocation scheme of division-of-work and compared the corresponding classification accuracies with common-work strategy’s.

**Approach:**

This study developed an electroencephalograph (EEG)-based cBCI which had six instructions related to six different motor imagery tasks (MI-cBCI), respectively. For the common-work strategy, all five subjects as a group had the same whole instruction set and they were required to conduct the same instruction at a time. For the division-of-work strategy, every subject’s instruction set was a subset of the whole one and different from each other. However, their union set was equal to the whole set. Based on the number of instructions in a subset, we divided the division-of-work strategy into four types, called “2 Tasks” … “5 Tasks.” To verify the effectiveness of these strategies, we employed EEG data collected from 19 subjects who independently performed six types of MI tasks to conduct the pseudo-online classification of MI-cBCI.

**Main results:**

Taking the number of tasks performed by one collaborator as the horizontal axis (two to six), the classification accuracy curve of MI-cBCI was mountain-like. The curve reached its peak at “4 Tasks,” which means each subset contained four instructions. It outperformed the common-work strategy (“6 Tasks”) in classification accuracy (**72.29 ± 4.43** vs. 58.53 ± 4.36%).

**Significance:**

The results demonstrate that our proposed task allocation strategy effectively enhanced the cBCI classification performance and reduced the individual workload.

## Introduction

Brain–computer interface (BCI) systems could use human brain signals for the direct control of external devices (Wang and Jung, 2011; Jiang et al., 2018). Compared with other ways of human machine interaction (HCI), such as voice or gesture (Karpov and Yusupov, 2018), BCI systems have the potential to provide more efficient HCI channels by encoding brain signals directly. It could express intended human actions and monitor human physiological states by detecting and analyzing neural activity. Brain–computer interface systems can be differentiated based on the brain-sensing modality employed, such as functional magnetic resonance imaging (fMRI) (Sokunbi et al., 2014), near infra-red spectroscopy (NIRS) (Naseer and Hong, 2015), and electroencephalography (EEG) (Abiri et al., 2019). Each of these modalities has certain advantages, which render it more suitable for specific applications. Due to the high time resolution and portability of EEG-based BCI, it is usually employed in the control of external devices (Luu et al., 2017; McCrimmon et al., 2018).

For control purposes, it can be divided into two types: (A) active BCI systems that do not require external stimuli which only use consciously intended brain signals. Motor imagery BCI (MI-BCI) is one of the mature representatives (Vourvopoulos et al., 2019; Zapała et al., 2020). (B) Reactive BCI systems are driven by indirectly modulated brain signals related to specific exxternal stimulation, such as steady-state visually evoked potential BCI (SSVEP-BCI) (Ma et al., 2017). However, most of them have not been widely used so far in social and productive activities mainly due to the following two reasons:

(1) Low information transmission rate: due to volume conduction effects of the brain, the EEG signal-to-noise ratio is relatively low (Liu, 2019; Wei et al., 2019). Hence, EEG-based BCI systems are generally incapable of extracting sufficiently effective neural features in a short time window, which results in poor decoding performance. On the other hand, for a high level of human–computer hybrid intelligence, elaborate control operations with high precision, short time delays, and long-term reliability are needed. These performance requirements are hardly met by current EEG-based BCI systems.
(2) Poor interpersonal collaboration: currently, the majority of BCI systems are designed for a single user, which are hard to meet the demands of social interactions and the large-scale collaboration of social groups. Human social interactions suggest that BCI systems should involve forms of collaboration with multiple persons and computers (Mattout, 2012).

To overcome the above limitations, collaborative BCI (cBCI) systems have been proposed. It is defined as BCIs where data from multiple users are integrated to achieve a common purpose (Valeriani et al., 2017). The classification performance and robustness could be effectively improved by fusing group EEG features. Therefore, cBCI systems are more suitable for advanced tasks of hybrid human–computer intelligence, especially group interactions (Valeriani et al., 2015).

Current cBCI systems can be divided into two categories based on their goals. One kind of cBCI systems is utilized to perform visual target matching or search tasks, which seeks to improve the system decision-making ability based on human visual information (Matran-Fernandez and Poli, 2014; Valeriani et al., 2015, 2017). The other kind of cBCI systems focuses on the output by movement intentions, which can carry out active control instructions much faster and more conveniently (Wang and Jung, 2011; Zhou et al., 2019). These studies show that BCI performance can be effectively improved by fusing the neural responses of multiple users for the same task. However, they did not explore how to design a better system architecture to achieve more efficient fusion of multiple sources of human brain information. We believe that two improvements are of vital importance in optimizing system design:

(1) Task allocation strategy: for existing cBCI systems, collaborators follow a common-work strategy, i.e., users perform the same task together. Nevertheless, this strategy does not fully consider the rationality of task allocation and the differences in individual capabilities. It may result in wasteful use of collaborative resources, without effectively improving the overall performance. By contrast, group performance might be improved through division-of-work strategy. In fact, Adam Smith, one of the key founders of free-market economics, suggested in his book “The Wealth of Nations” (Smith, 1848) that division-of-work greatly improves labor productivity. Hence, we designed the cBCI system with an optimizing task allocation strategy of division-of-work, in order to enhance the overall system performance and reduce the individual workload as well.
(2) Data-fusing method: Wang and Jung (2011) presented two paradigms of cBCI—centralized and distributed systems. The biggest distinction between the two is whether the brain information of multiple persons is processed centrally on one data server (centralization) or not (distribution). Different paradigms dictate distinct requirements of data fusion methods. Thus, we designed a *feature fusion method* for centralized paradigm which conducts unified modeling and recognition through integrating the EEG features of all collaborators. Besides, a *decision fusion method* was developed to compute an overall decision value of classification in the distributed paradigm. For the classification performance of cBCI, a comparison was undertaken between the two methods under multiple strategies of task allocation in this work.

Motor imagery is the mental representation of movement without any body movement (Dickstein and Deutsch, 2007). In our previous research (Zhou et al., 2019), a MI-cBCI system was successfully implemented by decoding event-related de-/synchronization (ERD/ERS) features from multiusers. This study still adopted the motor imagery paradigm, which is suitable for active control. Through the pseudo-online process of MI-cBCI, we explored the impact of two key factors: (1) task allocation strategy and (2) data fusion method on system classification performance.

## Materials and Methods

### Subjects

The study involved 19 healthy volunteering subjects (11 females, 23–27 years). None of these participants had cognitive or physical dysfunction. Nine subjects had previously participated in MI-BCI studies. The rest of the subjects had no BCI experience prior to this study. All participants read and signed the informed consent form approved by the Institutional Research Ethics Committee of Tianjin People’s Hospital before the experiment.

### Paradigm Design

In this study, we aim to address the problem of classifying six types of motor imagery instructions, namely, moving both hands (BH), both feet (BF), the left hand (LH), the right hand (RH), the right hand and the left foot (RHLF), and finally the left hand and the right foot (LHRF). Table 1 shows the details for these categories. For example, the name of the first type is “both hands.” Participants were required to perform MI of both wrist extensions. The command abbreviation is BH, the symbol is ↑ and the instruction number is 1. The motion associated with the foot task is ankle dorsiflexion.

**TABLE 1:** Categories of motor imagery instructions for the MI-cBCI system.

**Name**	**Both hands**	**Both feet**	**Left hand**	**Right hand**	**Right hand left foot**	**Left hand right foot**
Abbreviation	BH	BF	LH	RH	RHLF	LHRF
Diagram	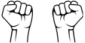	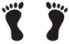	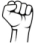	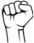	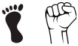	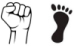
Symbol	↑	↓	←	→	↗	↘
No.	1	2	3	4	5	6

**FIGURE 1:**
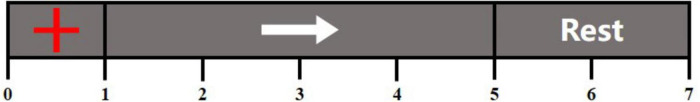
Experimental paradigm of a motor imagery task. At the beginning of each trial, a red fixation cross was presented at the center of the screen to remind subjects to prepare for the following task. At the first second, a symbol of instruction appeared on the screen for 4 s, subjects were instructed to perform the indicated motor imagery (MI) task up to the fifth second. This time period of 4 s was defined as a MI epoch. Then, “Rest” was displayed for 2 s to remind participants to have a rest.

All 19 subjects independently performed the above six types of motor imagery tasks with EEG data collected simultaneously. Then, the MI-cBCI system based on the division-of-work strategy was simulated by using offline EEG data from users. The whole experiment for a single subject was divided into 14 blocks, consisting of 36 trials (6 types × 6 trials) each, which led to 84 trials of each type of MI task. There was a break of about 5 min between the consecutive three blocks. Within each block, MI tasks were performed in a random order. The task paradigm is shown in Figure 1, which mainly includes a period of motor imagery that lasts 4 s. The experiment was programmed using Psychtoolbox on MATLAB platform.

### Data Acquisition and Preprocessing

The EEG signal was recorded using a SynAmps2 system (Neuroscan Inc., Charlotte, NC, United States) with a 64-channel quick-cap at a sampling rate of 1,000 Hz, whose electrode positioning follows the international 10/20 system. The reference and ground electrode were placed at the vertex and on the prefrontal lobe, respectively. A band-pass filter between 0.5 and 100 Hz and a 50-Hz notch filter were enabled during the data acquisition. All raw data were downsampled to 200 Hz and re-referenced by the common average reference (CAR). According to data labels, the EEG data of all trials were extracted as data samples. Then, data samples were band-pass filtered to obtain interested frequency (8–28 Hz) by a fourth-order Butterworth filter. All 84 samples of each class of MI are divided into two parts randomly. One part is for offline training, including 72 samples, and the other part includes 12 samples for the pseudo-online classification of cBCI.

### Algorithms

All the main algorithms applied in this study are described here, in order to avoid disrupting the continuity of the introduction of the overall workflow. The preprocessed EEG data collected from the motor imagery tasks were analyzed in the succeeding sections.

#### Event-Related Spectral Perturbation

Event-related spectral perturbation can provide detailed information about temporal and spatial ERD/ERS features of various MI categories (Yi et al., 2017). It is a useful tool to select the MI task with stronger feature separability from six instructions as the reference instruction, rather than for classification. The average event-related spectral perturbation (ERSP) across the input data is defined as follows:

(1) $$\begin{array}{c} \text{ERSP}\,\left(f,t\right)=\frac{1}{n}\,\sum_{k=1}^{n}\left(F_{k}\,{\left(f,t\right)}^{2}\right) \end{array}$$

where *n* is the number of trials, and *F*_*k*_(*f*,*t*) indicates the spectral estimation of the *k*th trial at frequency *f* and time *t*. To produce the baseline-normalized ERSP, the spectral estimation of a baseline period (1 s before the MI epoch) is subtracted from the ERSP of tasks. To observe time–frequency domain features, plots of the mean ERSP from two key electrodes C3 and C4 were displayed from -1 to 6 s between 8 and 28 Hz for analysis. To investigate the topographical distributions of ERD features, the average ERD values were computed within the specific frequency range and time window for each channel according to the following equation:

(2) $${\text{ERD}}_{\text{value}}=\frac{1}{N}\,\sum_{f\in F}\sum_{t\in T}\left(\mathrm{ERSP}\,\left(f,t\right)\right)$$

where *F* is the α band (8–13 Hz) or β band (14–25 Hz), and *T* is the whole MI task duration of 4 s. *N* is the total number of time–frequency bins decided by *F* and *T*.

#### Multiclass Common Spatial Patterns

Multiclass common spatial patterns (multi-CSP) was applied to extract features from multichannel EEG data of MI epochs (Qian et al., 2011; Yi et al., 2013). For the analysis, a single MI epoch data is represented as an *N* by *T* matrix X_*i*_, where *i*ϵ{1,2,…,6} indicates the *i*th class of MI, *N* is the number of channels (*N* = 60), and *T* is the number of samples per channel (*T* = 800). We firstly calculated the average covariance matrix *R*_*i*_of every MI pattern. The whitening matrix can be formed by

(3) $$P=\Lambda^{-1/2}\,U_{0}^{T}$$

where *U*_0_ is the *N* × *N* matrix of eigenvectors and Λ is the diagonal matrix of eigenvalues from

(4) $$R=\sum_{i=1}^{6}R_{i}=U_{0}\,\Lambda \,U_{0}^{T}$$

The strategy of one-versus-rest is adopted to acquire spatial filter matrices. For the first class, we let*R*_1_′=∑_6*i* = 2_*R*_i_. Then *R*_*1*_ and *R*_*1*_′ can be translated as

$$Y_{1}=P\,R_{1}\,P^{T}$$

(5) Y1′=P⁢R1⁢PT′

And *Y_1* and *Y_1*′ share common eigenvectors

$$Y_{1}=U_{1}\,\Lambda_{1}\,U_{1}^{T}$$

(6) Y1′=U1⁢Λ1⁢U1T′

With the projection matrix $W_{1}=U_{1}^{T}\,P$ consisting of spatial filters corresponding to the first class, the other five projection matrices also can be computed in a similar way.

#### Mutual Information Maximization

Mutual information maximization (MIM) (Khaleghi et al., 2015) was used in the feature fusion method to select features from the integrating features of all single users. The mutual information (MI) between every feature and its class label separately was calculated. Then features were ranked according to a decrease of MI. MI is defined as:

MI⁢(Y,X)=H⁢(Y)+H⁢(X)-H⁢(Y,X)

(7) $$=-\sum_{i,j}P\,\left(y_{i},x_{i}\right)\,{\text{log}}_{2}\,\frac{P\,\left(y_{i},x_{i}\right)}{P\,\left(y_{i}\right)\,P\,\left(x_{i}\right)}$$

where *H* function is the information theory,

(8) $$H\,\left(X\right)=-\sum_{i=1}^{K}P\,\left(x_{i}\right)\,{\text{log}}_{2}\,P\,\left(x_{i}\right)$$

(9) $$H\,\left(Y\right)=-\sum_{j=1}^{K}P\,\left(y_{i}\right)\,{\text{log}}_{2}\,P\,\left(y_{i}\right)$$

(10) $$H\,\left(Y,X\right)=-\sum_{i=1}^{K}\sum_{j=1}^{K}P\,\left(y_{i},x_{i}\right)\,{\text{log}}_{2}\,P\,\left(y_{i},x_{i}\right)$$

*P*(*x*_*i*_)and *P*(*y*_*i*_) are the *i*th priori probability of feature vector *X* and label *Y* in all *K* values, respectively. *P*(*y*_*i*_,*x*_*i*_) is the joint probability of them. After ranking the features, the first four features are reserved for processing in this work.

#### Multiclass Classification Support Vector Machines

Multiclass classification support vector machines (multi-class SVM) were employed to classify multiclass of features (Duan and Keerthi, 2005; Aboalayon et al., 2015). It constructs *M* binary classifiers, where *M* is the number of classes. Each classifier is trained to separate one class as positive from the rest of the *k* - 1 classes as negative.

Next, we describe in detail the concepts of task allocation and data processing flow in MI-cBCI systems.

#### Task Allocation Schemes Based on the Division-of-Work Strategy

**FIGURE 2:**
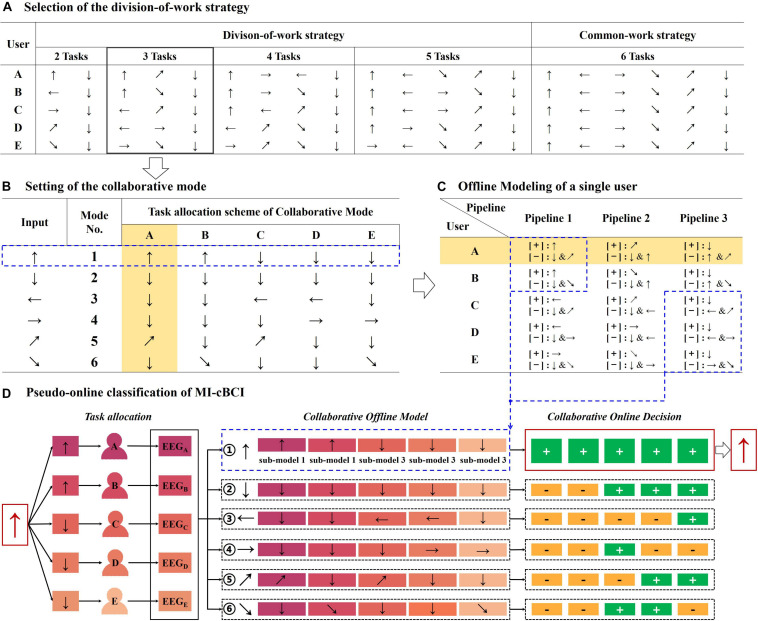
Workflow of the division-of-work strategy for the proposed MI-cBCI system. Arrows indicate different types of motor imagery instructions as shown in Table 1. [+]/[−] in “panel C” means taking the following instruction as a positive/negative class. +/− in “panel D” means a positive/negative decision label.

We propose an optimized task allocation scheme based on the division-of-work strategy for MI-cBCI systems. This strategy generates a feasible scheme to assign different MI tasks to collaborators. The MI-cBCI system has the same instruction set as a single-user MI-BCI system which has six MI instructions. A collaborative group consisted of five users, denoted by the letters A–E. In other words, the MI-cBCI system is operated by five persons controlling six instructions together. All of the users were randomly selected from 19 subjects. The workflow of the division-of-work strategy in MI-cBCI is divided into four steps:

(A) Selection of the division-of-work strategy. As shown in Figure 2A, there are four types of division-of-work strategies. Based on the size of the instruction subset for one person, they are categorized into “2 Tasks,” “3 Tasks,” and so on until “5 Tasks.” In addition, “6 Tasks” is the common-work strategy where each of the five users executes the identical six MI tasks. Both feet (BF) instruction is selected as the reference instruction that is involved in every single users’ instruction set. Here, we choose the “3 Tasks” strategy (in the solid black box) as an example to illustrate the following workflow.
(B) Setting of the collaborative mode. The input of the MI-cBCI system is defined as the required MI task, and the output is the instruction obtained by decoding the EEG information of all users. As shown in Figure 2B, six collaborative modes are set up to indicate the designated tasks to users in line with the input instructions. In most modes, two users are required to complete the tasks consistent with the system input, while others execute the both feet task. Only mode 2 requires all users to perform both feet tasks together. As an example, in the blue dashed box, the system input is ↑. According to the task allocation scheme of mode 1, users A and B should perform both hands MI (↑) and the remaining users perform both feet MI (↓).
(C) Offline modeling of a single user. Across all modes, each user executes a total of three types of tasks represented by arrows, which is in accordance with the “3 Tasks” strategy. Each arrow in Figure 2B matches a single-user offline modeling pipeline in Figure 2C. For instance, in the yellow shading area in Figure 2B, user A executes three kinds of MI tasks (↑↗↓). For these tasks, three data processing pipelines have been established, as shown in the yellow shading area in Figure 2C. Each pipeline is to complete the corresponding offline modeling of EEG data in the light of the one-versus-rest strategy. It means that one type of MI data is taken as the positive class [+], and the other two types of data became the negative class [−]. Features of two classes EEG data are extracted by the CSP algorithm and classified by a SVM classifier. In all pipelines of a single user, a total of three pairs of CSP filters and SVM classifiers have been trained. In the next offline phase of cBCI, they would be used as the submodels for the collaborative model. Detailed information about data processing of a single user have been described in the *Offline modeling of a single user* section.
(D) Pseudo-online classification. The pseudo-online classification of MI-cBCI is composed of two processes: the offline phase for cBCI modeling and the pseudo-online phase for recognition. In the offline phase, we established six collaborative models for feature extraction and classification, one for each of the collaborative modes in Figure 2B. For each collaborative model (①-⑥) in Figures 2B,D shows what submodels it should entail. Every collaborative model is assembled from five submodels. The submodels are generated by offline modeling of the users in step (C). Take the collaborative model ① as an example; it is set up with two submodels from pipeline 1 (users A and B) and three submodels from pipeline 3 (users C, D, and E), as shown in the blue dashed box in Figures 2C,D. Other collaborative models are built in the same way. There are two alternative fusion methods applied in constructing collaborative models called feature fusion and decision fusion, which are described in the *Feature fusion method* and *Decision fusion method* sections.

In the pseudo-online phase, EEG data collected from five collaborators are sent to the six collaborative models sequentially for classification. The collaboration model that has the highest number of submodels matched to the multiperson input data is the winner, and its corresponding mode (i.e., the arrow that immediately follows numbers ①-⑥ in Figure 2D) is selected as the final system output. To illustrate the process of pseudo-online recognition more specifically, we take the system input of ↑ as an example in Figure 2D. It shows that users A and B need to imagine both hands (↑) while the other users are required to imagine both feet (↓) as defined in the task allocation scheme in Figure 2B. Subsequently, pseudo-online EEG data from all five users (marked as different colors) are processed by the six collaborative models in sequence. Because the input EEG data match to the positive classes of all five submodels of collaborative model ①, it should contain the largest number of positive decision labels among all six collaborative models. Therefore, the system output is both hands instruction (↑).

### Data Processing of MI-cBCI

After describing the overall workflow, we will concentrate on the details of data processing. Two data fusion methods for MI-cBCI have been proposed in this study, which are called feature fusion and decision fusion. The implementation of both methods is based on the single-user modeling process and differed on the subsequent cBCI offline modeling and pseudo-online validation.

#### Offline Modeling of a Single User

**FIGURE 3:**
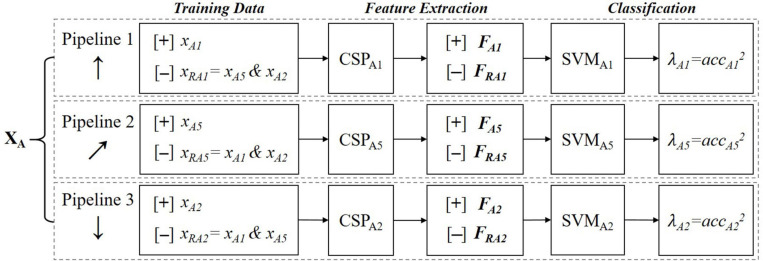
The data processing procedure of a single user for offline modeling. *X*_*A*_ represents the training dataset of subject A. *x* means a certain class of data. [+]/[−] means taking the following data as a positive/negative class. CSP and SVM indicate CSP filters and SVM classifiers, respectively. We use the symbol ***F*** to represent the feature matrix. acc is the abbreviation of accuracy.

The purpose of single-person offline modeling is to provide the required submodels for cBCI, mainly including CSP filters and SVM classifiers. Here, we continue with the example of the “3 Tasks” strategy. According to the task allocation scheme in Figure 2C, subject A should perform BH, BF, and RHLF tasks (numbered 1, 5, and 2, respectively). Figure 3 illustrates the offline modeling process of EEG data related to these instructions. Each class of EEG data is taken in turn as a positive class [+], while others represent the negative class [−]. All three data processing pipelines are made to accomplish binary classifications. For example, in pipeline 1, *x*_*A1*_ represents the data of executing instruction 1 and *x*_*RA1*_ represents two remaining data (*x*_*A5*_ and *x*_*A2*_). Feature matrices **FA1***ϵℝ*^72×4^ and **FRA1***ϵℝ*^144×4^are obtained by filtering 72 samples of *x*_*A1*_ and 144 samples of *x*_*RA1*_, respectively. A SVM classifier with linear kernel function is trained using the two classes of features. Leave-one-out cross-validation is applied to obtain the offline accuracy acc_*A1*_, and an accuracy-based weight coefficient λ_A1_ = acc_*A1*_^2^ is computed to guide subsequent offline processing of cBCI.

#### Feature Fusion Method

**FIGURE 4:**
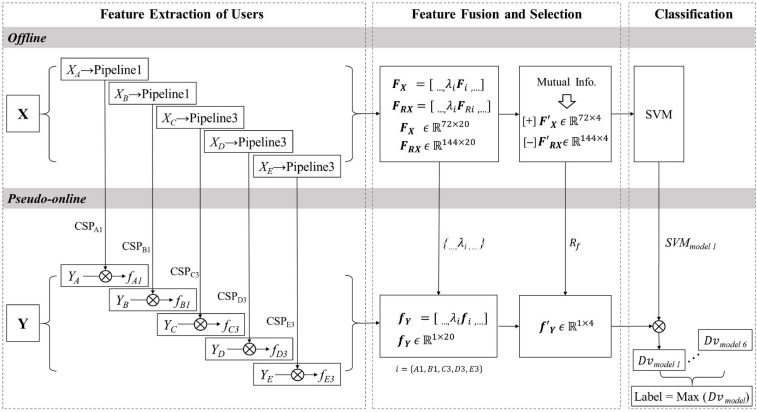
The data processing procedure for the feature fusion method. 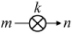 means that *m* is processed by component *k* (a filter or a classifier) to obtain data *n*. Mutual Info and Dv are the abbreviations of mutual information and decision value, respectively.

Figure 4 demonstrates the data processing procedure of the feature fusion method for MI-cBCI. In the multiperson cBCI offline phase, **X** represents the EEG training dataset for five collaborators. The selection of data processing pipelines of users depends on the collaborative modes. We describe here the offline and pseudo-online process of mode 1. The data processing pipeline of each user is executed independently using training datasets. As described in the previous section, all submodels containing feature matrices of the two classes **Fi** and **F**_**R***i*_, CSP filters, and weight coefficients λ_*i*_ are all obtained from five collaborators, *i* = {A1,B1,C3,D3,E3}. **Fi***ϵℝ*^72×4^ and **F**_**R***i*_*ϵℝ*^144×4^ are multiplied by their respective coefficients λ_*i*_ and concatenated into matrices **FX***ϵℝ*^72×20^ and **F**_**R***X*_*ϵℝ*^144×20^ in the column direction. After that, the features are sorted in descending order by the mutual information criterion, and the achieved feature ranking *R*_*f*_ is recorded. Only the top 4 features are pick up as F⁢ϵ′X⁢ℝ72×4 and F⁢ϵ′R⁢X⁢ℝ144×4, respectively. Finally, a SVM classifier is trained for offline modeling of mode 1 by taking FX′ and FR⁢X′ as positive/negative class.

In the cBCI pseudo-online phase, ***Y*** contains single-trial data extracted from five users’ testing dataset. The CSP filters from the offline modeling phase are applied to filter ***Y*** to calculate single-user features **fi***ϵℝ*^1×4^. Then, the multiuser features are combined (following the offline processing approach) to gain the selected features **fY***ϵℝ*^1×20^. According to the feature ranking *R*_*f*_, the fusing features are rearranged and the first four features are selected as f⁢ϵ′Y⁢ℝ1×4. Then, the optimized features are classified by the trained classifier SVM_*model 1*_ to export the decision value Dv_*model 1*_. Using the same method, we process ***Y*** with the other five models and subsequently acquire the outputs Dv_*model 2*_… Dv_*model 6*_. The label associated with the maximum positive decision value is considered to be the predicted label.

#### Decision Fusion Method

**FIGURE 5:**
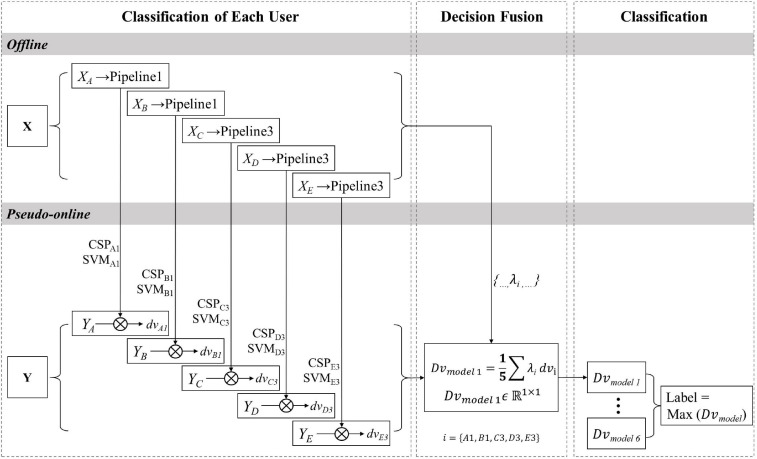
The data processing procedure for the decision fusion method.

Figure 5 shows the data processing procedure of the decision fusion method for MI-cBCI. It fuses information on the decision value level, while the feature fusion method is on the feature level. Specifically, in the offline phase, the training dataset X is processed with different pipelines from collaborators. The CSP filters and SVM classifiers are reserved, and the accuracy-based weights λ_*i*_ are also calculated. In the pseudo-online phase, multiple pairs of CSP filters and SVM classifiers are utilized to conduct spatial filtering and classification on the multiusers’ EEG testing data. The corresponding decision value vector dv*iϵℝ*^1×1^ is calculated. In addition, decision values from multiple users are fused to get the decision value vector of model 1 Dvmodel⁢ 1=15⁢∑λi⁢dvi. In turn, the output value Dv_model_ of each model is calculated, and then the label corresponding to the maximum positive value is chosen as the predicted label.

## Results

### Event-Related Spectral Perturbation

**FIGURE 6:**
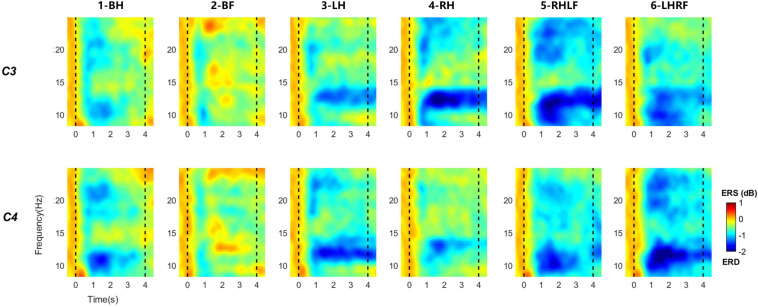
Averaged time–frequency maps across 19 subjects for six types of MI tasks at the location of C3 and C4 electrodes. Blue indicates ERD; red indicates ERS. Black dashed line indicates the onset and offset of motor imagery.

The C3 and C4 electrodes are located in the sensorimotor cortex of the brain (Li et al., 2019). As preliminary knowledge, they are the primary electrodes for the neural response features induced by MI (Tangwiriyasakul et al., 2013). Figure 6 shows the averaged ERSP time–frequency maps of two electrodes across 19 subjects performing six types of MI tasks. The two black dotted lines at time points 0 and 4 represent the start and stop time of MI, respectively. The color bar from blue to red represents the energy ratio from low to high compared with the baseline energy. The map presents clear spectral powers of ERD at α (8–13 Hz) and β (14–28 Hz) bands under various MI tasks. They last until the end of the MI task phase, especially for instruction 3-LH, 4-RH, 5-RHLF, and 6-LHRF. The ERD on both feet is the weakest, as shown in Figure 6. It also could be seen that the ERD in the α band is more obvious, and it has laterality with different instructions. In order to explore spatial distribution, averaged topographical maps of ERD are drawn in this study as well.

**FIGURE 7:**
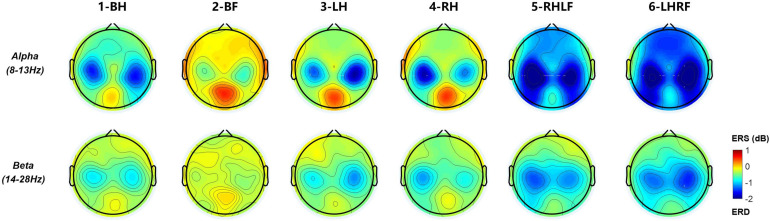
Averaged topographical distribution for six types of MI tasks at α (8–13 Hz) and β (14–28 Hz) bands. Blue regions indicate the involved areas where ERD occurs during the MI period.

Figure 7 is the average topographic map of all 19 subjects, and α (the first row) and β (the second row) bands for the MI period (4 s) are selected. It can be clearly observed that the ERD of unilateral upper limb MI has obvious contralateral dominance. Both hands’ movement induces marked enhancement of ERD on both sides. As we can view in the second column, the ERD of both feet is the weakest in the six types of MI, which is consistent with the time–frequency plot. The compound MI composed of one hand and one foot had significant ERD enhancement on both sides. Moreover, the whole brain has more significant energy attenuation than other types of MI. By contrast, the contralateral activation of the hand is stronger than that of foot MI, and the activation area is larger.

By superimposing the averages of multiple trials of ERSP, we can find that ERD features of 19 participants are actually induced in general, and the ERD of six types of MI is mainly located in the α and β bands with contralateral dominance, which is consistent with the results of previous studies (Sollfrank et al., 2015; Collazos-Huertas et al., 2020). Among the six types of MI tasks, the ERD of both feet MI task is the weakest, which could have the largest difference from other tasks. This is the reason why we chose it as a reference instruction.

### Classification Performance

**FIGURE 8:**
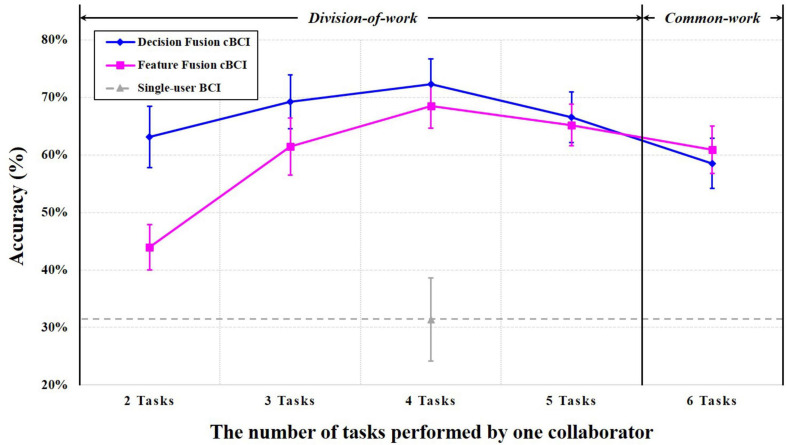
Classification accuracy curves of the feature and decision fusion methods for cBCI and single-user BCI.

In this work, we collected EEG data from 19 subjects who independently performed the abovementioned six types of MI tasks. We should select five persons as users A–E from 19 subjects to conduct the pseudo-online classification of MI-cBCI. The maximum number of possible selections is the number of five permutations of 19. To reduce the complexity, we randomly picked 300 selections among them, and the average classification accuracy of MI-cBCI was obtained for simulated online classification, as shown in Figure 8. The vertical coordinate shows the average classification accuracy of six instructions, and the horizontal coordinate represents the number of tasks performed by one collaborator; “2 Tasks” to “5 Tasks” belong to the division-of-work strategy, while the “6 Tasks” strategy is the conventional common-work strategy. The classification accuracies of the cBCI systems using feature fusion and decision fusion methods are depicted by the pink and blue lines, respectively. The gray dotted line shows the six-class average classification accuracy of 19 subjects by the single-user BCI system. This accuracy is independent of the task allocation and does not change with the horizontal coordinate.

These results show the following: (1) even at the lowest point of the cBCI performance curves, the cBCI average classification accuracy is more than 10% higher than the single-user BCI (feature fusion cBCI at “2 Tasks”: 43.97 ± 3.96%, decision fusion cBCI at “6 Tasks”: 58.53 ± 4.36%, single-user BCI: 31.37 ± 7.21%); (2) accuracy peaks of both classification curves are at “4 Tasks” (division-of-work), which is higher than “6 Tasks” (common work): feature fusion cBCI (68.48 ± 3.85 vs. 60.93 ± 4.13%) and decision fusion cBCI (72.29 ± 4.43 vs. 58.53 ± 4.36%); (3) comparison of the cBCI performance curves indicates the superiority of the decision fusion cBCI system over the feature fusion for most of the division-of-work strategies; (4) the standard deviation of the classification accuracy is reasonably small which almost remains within 5%. This low standard deviation shows that the subject selection may have little impact on the cBCI system performance. Therefore, these results obtained by randomly selected users are representative and authentic.

## Conclusion and Discussion

In this work, a novel task allocation based on division-of-work strategy for MI-cBCI system is proposed. The recognition performance metrics indicated that the division-of-work systems outperform the common-work system, and showed better accuracy than the single-user BCI system. We believe that the main reason for this is due to that division-of-work strategy effectively reduced the number of classes in multiclassification for single person, thereby improving the accuracy of it. Generally speaking, the classification performance of cBCI is positively correlated to the single-person performance and the number of users executing the common tasks. Although the division-of-work strategy reduces the number of users recognizing the same instructions, it improves the classification performance of a single person. The influences of these two factors on the system are the reasons why the shape of the classification accuracy curves are mountain-like in both methods. In the current system, the accuracy peak is at the “4 Tasks” strategy.

Moreover, this paper compares the recognition performance of two data-fusing methods and shows that the decision fusion algorithm is generally superior to the feature fusion. Currently, the literature suffers from the lack of extensive discussions on this problem. We are aware of little relevant work on this problem, except for the cBCI based on rapid serial visual presentation (RSVP) which was designed by Matran-Fernandez and Poli (2014). Moreover, they came to similar conclusions to ours in spite of the employment of different potential features.

We are here to discuss the reasons for the difference in performance between the two methods. Specifically, decision fusion for distributed architecture is more direct, while feature fusion for centralized architecture retains more EEG information of individuals and may lead to degraded performance. If more efficient multiperson EEG feature extraction algorithms can be applied, e.g., algorithms based on deep learning or transfer learning, feature fusion cBCI could capture more relevant information and may thus have greater research potential.

We believe that future cBCI research should have more hybridization and collaboration in the following aspects: (1) *hybrid tasks*: the current cBCI tasks are usually single tasks, which are basically enhancement tasks for motion control or visual recognition (Liu et al., 2020). However, cBCI systems may perform better in hybrid high-load tasks and have greater advantages of collaboration; (2) *Joint task allocation strategies and data-fusing methods*: more tasks lead to inferior performance under a standalone task allocation scheme. Therefore, cBCI systems should be adjusted continuously according to operation characteristics and user capabilities. More specifically, cBCI systems may be created with hybrid common-work and division-of-work strategies, as well as hybrids of centralized and distributed architectures. It can assign dynamic specific tasks and data processing methods according to the status of each collaborator; (3) *fusion of multimodal signals*: multiple neural response features (e.g., potential and energy) should be deeply mined and fused (Wang et al., 2020). Also, cBCI systems with other physiological or behavioral signals might be exploited. Furthermore, fusion strategies can be adjusted to achieve automatic performance optimization.

On one hand, the development of the cBCI technology indicates that the performance of existing BCI systems can be substantially improved. On the other hand, cBCI technology evolution promises the development of a new generation of human–computer interaction systems with energy-saving and networking modes. In addition to the abovementioned cBCI systems, passive cBCI systems whose operation is based on monitoring the interaction between multiple persons and the external environment have been gradually emerging. This technology is also known as hyperscanning. In recent years, hyperscanning systems based on EEG, functional near-infrared spectroscopy (fNIRS), and magnetoencephalography (MEG) have been rapidly developed. Through designing joint tasks to explore the brain activation characteristics and causality (Konvalinka and Roepstorff, 2012; Sänger et al., 2012; Babiloni and Astolfi, 2014; Nam et al., 2020), the conventional interaction between individual subjects, tasks, and the environment has been gradually transformed into the interaction between multiple persons, multiple tasks, and different environments. Hence, the cBCI technology is expected to spread more widely and be more successful in novel and diverse engineering applications.

## Data Availability Statement

The raw data supporting the conclusions of this article will be made available by the authors, without undue reservation.

## Ethics Statement

The studies involving human participants were reviewed and approved by the institutional research ethics committee of Tianjin People’s Hospital. The patients/participants provided their written informed consent to participate in this study.

## Author Contributions

BG and MX designed the study. BG, LC, KW, and JT performed research. BG and MX analyzed data. BG, YK, MX, LX, and DM wrote the manuscript. All authors contributed to the article and approved the submitted version.

## Conflict of Interest

The authors declare that the research was conducted in the absence of any commercial or financial relationships that could be construed as a potential conflict of interest.
